# Lessons from a community based interdisciplinary learning exposure: benefits for both students and communities in Uganda

**DOI:** 10.1186/s12909-020-02429-2

**Published:** 2021-01-04

**Authors:** Esther Buregyeya, Edwinah Atusingwize, Peninah Nsamba, Christine Nalwadda, Jimmy Osuret, Patrick Kalibala, Ronald Nuwamanya, Samuel Okech, Tonny Ssekamatte, Sarah Nitumusiima, Timothy Wakabi, Winnie Bikaako, Agnes Yawe, Irene Naigaga, Juvenal Kagarama, John David Kabasa, William Bazeyo

**Affiliations:** 1 Makerere University School of Public Health, Kampala, Uganda; 2 Makerere University College of Veterinary Medicine, Animal Resources and Biosecurity, Kampala, Uganda; 3 One Health Central and Eastern Africa (OHCEA), Kampala, Uganda

**Keywords:** One Health Institute; community based training, Multi-disciplinary field attachment, One Health approach, Uganda

## Abstract

**Background:**

Makerere University implemented a One Health Institute (OHI) in 2016 involving undergraduate students selected from different disciplines. The students were first taken through theoretical principles of One Health followed by a field attachment in communities. The field attachment aimed to expose students to experiential educational opportunities in the communities in a One Health approach. In this paper, we present students’ experiences and their contributions to the communities of attachment.

**Methods:**

This was a cross-sectional study, utilizing qualitative data collection methods. The study involved students who participated in the OHI field attachment and community members in a One Health demonstration site-Western Uganda. Four focus group discussions (FGDs) and four in-depths interviews (IDIs) were conducted among the students, while four FGDs and twelve IDIs were conducted among community members. All interviews were audio-recorded, transcribed and analysed manually.

**Results:**

The four themes that emerged are: students’ understanding and appreciation of One Health concept, their experiences and gains from the multi-disciplinary field attachment, students’ contributions to the community, and challenges faced by the students. Students had good knowledge of One Health. They appreciated that health cannot be achieved by one discipline or sector and thus the need to collaborate across sectors. Regarding experiences and gains during the multi-disciplinary field attachment, the students appreciated that each discipline had a role to play in achieving health in the community. They appreciated the training citing skills gained in communication, team work and collaboration. They also reported a feeling of gratitude and accomplishment because they felt they made a positive change to the community by putting in place interventions to address some of the community challenges. Similarly, the communities appreciated the students’ contribution in solving their health challenges, ranging from conducting health education to improving sanitation and hygiene.

**Conclusions:**

Through the OHI, students gained One Health competencies including communication, teamwork, and collaboration. Adopting an interdisciplinary model in university teaching system especially during field placement would strengthen skills of collaboration, team work and communication which are critical for a multi-disciplinary approach which is needed among the future workforce in order to solve the current health challenges.

## Background

The beginning of the twenty-first century has been marked by many global public health challenges. Inequities in health persist both within and between countries, emphasizing the failure to share health advances fairly [1]. At the same time, new health challenges have emerged such as new infectious diseases, environmental and behavioural risks, rapid demographic and epidemiological transitions threatening health security of all [1]. Solving these complex global health problems requires innovation and a paradigm shift in the traditional systems for response with a deep understanding of the animal–human–ecosystem interface [2, 3]. Globally, the multi-sectoral and inter-disciplinary mode or the One Health approach is currently being advocated for [4]. One Health is the collaborative efforts of multiple disciplines working locally, nationally, and globally, to attain optimal health for people, domestic animals, wildlife, plants, and the environment [4]. It is a conceptual extension of traditional public health approaches, and methodologies because it expands to link human, animal, and wildlife health with the environment to address the risks of new health challenges such as pandemics of emerging and re-emerging diseases, climate change and pollution [5]. The concept has been recognized to hold promise in solving current health challenges and fostering optimal health for people, animals and the environment [6–8]. When properly implemented, One Health approach is perceived as a means to realize the United Nations Sustainable Development Goals and will help protect and save millions of lives in the current and future generations [9]. Therefore, the added value of One Health approach is; through improved sustainability of impact (One Health approaches should aim to produce positive outcomes that are resilient, productive, and endure over time), increased cost-effectiveness, and the enhanced potential for mitigating harmful outcomes [10].

Several factors have changed interactions between people, animals, and environment leading to the emergence and re-emergence of many diseases. For example, over 60% of emerging or re-emerging diseases have animal origins especially wildlife. In addition, at least 70% of the known human and animal pathogens affecting public health, global trade, and security are resident in Sub-Saharan Africa and in particular, Eastern and Central Africa [11, 12]. Uganda in particular is considered a ‘hot spot’ for emerging and re-emerging infectious diseases [13]. Thus, successful public health interventions in Uganda and the surrounding region require the cooperation and collaboration of human, animal, and environmental health sectors in order to attain optimal health outcomes for the environment, people and animals [14–16]. To achieve this, current silos must be broken down among clinical, biomedical, infectious disease, social sciences, population health, policy, and environmental health research [3]. Therefore, academic institutions should play a foundational role in breaking down these silos and facilitating transdisciplinary education and research initiatives [10]. However, health professional education has not kept pace with the current health challenges, largely due to fragmented, outdated, and static curricula that produce ill-equipped graduates [1]. Global movements and the Commission on the Education for health professionals for the twenty-first century have argued for a redesign of health professional education systems to better match current health challenges [1]. In order to make the future workforce be able to collaborate across sectors, appreciate each discipline’s contribution to health, and work in effective teams, they need to be trained in a multidisciplinary and professional interdependence manner [1]. In addition, Stroud and colleagues in their review on OH training in North America argued that it is vital to give students the chance to form relationships across disciplines very early in their training as these relationships will follow them throughout their careers, increasing their future comfort for working across professions [17]. Some of the institutional capacities that have been identified as important for facilitating One Health/transdisciplinary training and research include: (1) the promotion of transdisciplinary education opportunities that include community based methods, effective communication, and leadership; and (2) reduction in conflict and perceived power differentials among disciplines [16, 18]. Relatedly, community based education is important because it integrates education and practice in the learning process [19] to foster competency acquisition. It creates appropriate knowledge and attitudes; to promote social skills and multidisciplinary teamwork; and deepens trainees’ understanding of the contribution of social and environmental factors to causation and prevention of ill-health [20]. Further, inclusion of community stakeholders contributes to the adoption and sustainability of One Health initiatives [11, 21].

The training of future workforce in One Health is critical in order to improve the health and well-being of humans, animals, and ecosystems, thus promoting sustainable health for prosperous communities, productive animals, and balanced ecosystems. In a study exploring challenges in implementing an integrated One Health Disease Surveillance in Australia, developing interdisciplinary training in One Health concepts for medical, environmental and veterinary students was felt to encourage cross-disciplinary collaboration [22]. Since 2016, the US Agency for International Development (USAID) One Health workforce project (OHWF) has focused on developing a workforce with skills and mindset to tackle One Health challenges, including emerging infectious disease and antimicrobial resistance [23]. The One Health Central and Eastern Africa (OHCEA)- now AFROHUN, which is a network of 23 schools of veterinary and public health are championing the One Health in academia in Africa, by piloting an interdisciplinary training, with an aim to produce a One Health workforce. This is part of the USAID Emerging Pandemic Threats (EPT) Program designed according to a One Health model, in partnership with US and international universities, to train current and future One Health professionals [23]. Through OHCEA/OHWF, the Makerere University School of Public Health and College of Veterinary Medicine, Animal Resources and Bio-security jointly with other colleges at Makerere University designed and implemented an innovative One Health Institute (OHI) in 2016. The OHI is intended to train and transform the knowledge of young interdisciplinary and cross-sectoral teams of professionals at the start of their careers and give them ability to detect, prevent and respond to infectious diseases. At the time of this study, the OHI had run three cohorts of undergraduates (69 students in 2016, 30 in 2017 and 52 in 2018) from different disciplines since 2016. However, there is a dearth of information regarding how students specifically gain from the training and how they contribute to communities (where they do their practical attachment) during their field-based training. This study was conducted to explore the students’ gains, experiences of the OHI including their contributions to solving communities’ challenges.

## Methods

### Setting

The study was conducted in One Health field demonstration sites (Bwera and Kasese) in western Uganda; a country situated in the Congo Basin a ‘hot spot’ of zoonotic disease out breaks. This region has recently faced many outbreaks of zoonotic diseases including, rift valley and anthrax, Marburg, avian influenza and ebola (with current outbreak in the Democratic Republic of Congo) [13]. There is therefore urgent need both within Uganda and the neighboring region (Eastern and Central Africa) to develop or improve capacity to prevent, detect and respond to these infectious disease outbreaks using the One Health approach. The OHI is intended to train and transform the knowledge of young interdisciplinary and cross-sectoral teams of professionals at the start of their careers and give them ability to detect, prevent and respond to infectious diseases. The specific aims of the OHI are to equip students (future workforce) with competences to; i) be able to work in a multi-disciplinary teams, ii) respond to the community threats that are infectious or have the potential to be infectious using the One Health and iii) to effectively exchange information, and offer advice to the experts and community who face the threats that are infectious or have the potential to be infectious. Selected from the different disciplines at Makerere University through a competitive process, undergraduate students from final years of different disciplines are trained in a certificate course in principles of infectious disease management using One Health approach in order to prepare them to more effectively combat the spread of diseases in an environment they work and form relationships across disciplines, thus break the silos and facilitate collaboration. At the time of this study, since 2016, the OHI had run three cohorts of 151 undergraduate students (69 students in 2016, 30 in 2017 and 52 in 2018). The students included final students on the bachelor’s degree courses of human medicine and nursing [11]; environmental health [21], veterinary medicine, animal production and wildlife management [14]; biomedical laboratory sciences [17]; agricultural and forestry sciences, including engineering (20); social sciences, education, laws and the liberal arts [24]; and other degree courses like engineering, metrology, biological sciences, commerce, accounting, computer engineering [25]. Students are first taken through a 16-days didactic One Health seven courses including leadership, policy analysis, gender risk analysis, outbreak investigation, antimicrobial resistance, biosafety and bio-risk management and community entry and engagement. Following the theory component is a 28-days field attachment (in One Health field demonstration site), designed to expose students to experiential education opportunities in which the students get a chance to apply skills and knowledge gained. This mode of learning is aimed to enhance long term learning outcomes through students working closely with communities to identify their health threats by creating appropriate knowledge and attitudes; as well as promoting social skills and multidisciplinary teamwork [10] as well as foster adoption and sustainability [11].

Part of the preparation to ensure safety of the students during field attachment involves vaccination against rabies as well as giving them personal protective gear such as gum boots, gloves and clinical coats. In addition, students are oriented in general infection control measures during the course in biosafety and bio-risk management, mentioned above, which orients them in general infection control measures, such as hand washing, wearing protective gear when interacting with infectious substances. Briefly, the field experiential learning involves; i) 3-days of interacting with community leaders to explain the mission at hand and gain acceptance and confidence to work in a selected community. ii) This is followed by rapid assessment of the community for health challenges (7 days), working with communities to identify what they consider to be a problem/s. iii) Then a discussion (student-led) with the communities on potential interventions. Attempts are made to implement some simple and sustainable solutions to some challenges identified in consultation with the community (14 days). While in the field, students in study groups of approximately 14–15 students each are mentored by a multi-disciplinary team of faculty and field-based supervisors (including the district health officers, the district veterinary officers, the district environmental officers), with the help of the local councillors or village/ community leaders. Through community based training, students are expected to gain One Health competencies including team work, negotiation, collaboration and cooperation. During the final days of the field engagement, students make presentations of what they have done in an audience of the district officials and community members; as a way of accountability to the community, ensuring sustainability and ownership of interventions as well equipping them with communication skills.

### Study design, population and sites

This was an explorative cross-sectional study, utilizing qualitative data collection methods involving focus group discussions (FGDs) and key informant interviews (KIIs). The study participants included OHI students, students’ field-based mentors, members and leaders in communities of Kasese town and Bwera municipality in Kasese district, Western Uganda (where students had their community placement). FGDs and KIIs were conducted in communities where the students worked such as schools, markets, health facilities and abattoirs. Community members were selected to participate in the FGDs and the KIIs were for community leaders, managers of the selected places and mentors of students during the students’ field attachment (including the district health officers, the district veterinary officers, and local councillors). The Coordinator of the OHI selected the student respondents, while community leaders helped identify community respondents.

### Data collection

Data were collected in January 2018. Using English and local languages, a total of sixteen IDIs were conducted. These included; 1 male owner of an abattoir, 1 male laboratory technologist, 1 male senior clinical officer, 1 male town clerk, 2 veterinary male doctors, 1female district production officer, 1 male disease surveillance officer, 2 male local council leaders and 2 female village health workers. Four IDIs were conducted with student leaders in English. Four FGDs were conducted among community members in local languages, while four FGDs (two with science, and two with Arts students) were conducted in English, Table 1. Data were collected using interview guides developed by the research team focusing around the students’ understanding of One Health concept, their experiences and contribution towards solving community health challenges in the perspective of community members. We also elicited respondents’ views on how best can field attachment be organized and run so that it profits both the students and the community. Semi-structured guides were developed with follow-on questions or probes were used accordingly. Data were collected by research assistants with experience in qualitative research, who were re-trained on FGDs and IDIs, ethical conduct and study objectives by the research team (EB, EA and CN) with formal training and vast experience in doing qualitative research. All the interviews were audio recorded.

**Table 1:** Summary of the interviews

SN	Category of interview	Type	Number of interviews carried out
01	Science students	FGD	2
02	Art students	FGD	2
03	Student leaders	IDI	4
04	Community	FGD	4
05	Community	IDI	12
	**Total number of interviews**		**24**

### Data management and analysis

All interviews were transcribed verbatim (and translation to English for the FGDs that were conducted in the local language). The transcripts were manually analyzed, using the thematic content analysis. After reading transcripts several times by different research team members (mainly by EB, ET and CN), codes were agreed upon, and were used to code all the transcripts. Through consensus, related codes were merged into categories, which later were emerged into themes.

## Results

A total of 24 interviews was conducted and this was determined by saturation i.e. when no more new information came up. Four themes emerged including: students’ understanding and appreciation of One Health concept, students’ experiences and gains from the field attachment, students’ contributions to the communities and challenges faced.

### Students understanding and appreciation of one health concept

When asked about their understanding of One Health approach, all the students had good knowledge about the concept. They explained that it is how different disciplines work in a collaborative manner to address complex challenges. They recognized and emphasized that health cannot be achieved by one discipline or sector, due to the interdependence between human, animal and the environment. Thus, the need for collaboration and combined efforts as the corner stone to address the current health challenges.

*“My view about One Health is that it addresses human, animal and environment challenges, and this cannot be addressed by only one discipline, so knowledge cannot come from only one area. There is need to bring together ideas, knowledge and skills of different aspects and from different academic backgrounds to be able to solve these problems effectively.”* (IDI Student leader)

“…..*usually in reference to health and environment it is hard for one discipline, ok I have seen it, and now I can say that it is hard for one discipline to get a solution, it is more important to get ideas or approaches from different people so that you can get a good solution for that problem, either to get rid of it for once and for all or to stop it for some good time because as you can see some of these problems keep on coming back but getting a good solution deserve so many ideas from so many disciplines”.* (FGD Art students)

Students mentioned that sustainable health solutions can be best developed and implemented through multidisciplinary efforts of various stakeholders at different levels, including communities where public health challenges exist.

***“***...*, there should also be some bit of collaboration and partnership not only from people of the different disciplines but also from different leaders and different levels from within the community to ensure that you can be able to address those challenges in such a way that there is some bit of sustainability****.”*** (FGD Science Students)

In addition, students noted that One Health approach is different from the traditional way of solving health challenges, because it involves every discipline/sector which is critical for sustainable health. Recognizing lack of awareness about the concept and its importance in many settings, students highlighted a need for sensitization to raise awareness among stakeholders across various sectors for it to be embraced.

“*Well, One Health is not a traditional approach, it is a new system and handling that has been developed, it is an innovation that has come up to create a collective responsibility in problem solving. Traditionally we have been relying on the medical personnel only in case of an outbreak. Anthrax has occurred,…, brucellosis has occurred, and you look at a veterinary officer only, but now with One Health, we look at …what is the role of a farmer, what is the role of a market vender, then a vet who is coming to inspect meat at the slaughter house so it requires everyone’s input. This approach seems to be effective; we must market it, sensitize people about it, to get everyone on board and the system will be effective*.” (FGD Science Students)

Some students viewed the One Health approach as being an efficient method of solving challenges by creating a sense of responsibility and saving time for example in responding to an outbreak.

*“It is also another way of getting things done in the field within the shortest time possible because I am imagining he is a wildlife professional at the protected area which is faced with issues of social challenges. I see it can really be hard for him [wildlife professional] to think of social matters, related economics. But now if you integrate and bring in all those other people [social workers] concerned especially with people, work can be done in a very simple way and in a short time.* (IDI Student Leader)

### Students’ experiences and gains during the field attachment

Students’ experiences and gains were described in a number of ways as shown in the following categories; a) appreciation of the concept of team work, b) appreciation of the role of community involvement and c) innovative use of available resources.

- *Students’ appreciation of the concept of team work.*

Students appreciated the different disciplines working together to develop solutions to the challenges they identified in the communities. This was reported to be critical due to the complex nature of the interconnectedness of human, animal and the environment, and through team work they recognized that each discipline contributes importantly in achieving community health

*“You realize when humans are affected, most times it is connected with either the environment they stay in or the animals they stay with, and the reverse can be true, when the animals are affected, sometimes it is connected to the humans with those diseases…so going in depth in knowing all these causes, teasing out relationships, is one good reason for bringing in multi- disciplinary teams because when you collect ideas from different people, you can come up with one good reason as to why something is happening then you can figure out how to solve that problem.” (*FGD Arts Students)

*“These challenges are complex in nature... If you look at the general community, you will see that there are different aspects that are also being affected indirectly. If you concentrate at the health part of it you will affect the environmental part of it, the economical bit of it, the social being of the people living in that community will also be affected so if you try to bring in different groups of people on one table, and you ensure that all those different aspects are captured, it will help you to come up with an intervention or a solution whereby each group of people is more comfortable with and you know that once you take this direction this challenge will be worked upon.”* (FGD Science Students)

Other skills that were reportedly learnt included negotiation, stakeholder engagement, conflict resolution, and respect of other people’s opinions in order to foster effective team work and enhance integrated approaches. They noted that engagement with people of different background, social standards and professions requires collaborative efforts focused on respect of other people’s views.

*“… I learnt that it was good to engage people of different backgrounds, different levels of standards of living and people of different careers to ensure that you have the same goals. Another skill is on vision integration, because everyone comes with his/her own ideas and goals… so you try to bring all of them together. I also learnt diplomacy of solving conflicts within the groups, some of these conflicts you can’t avoid them.* (FGD Science Students)

b) *Appreciated the role of community involvement.*

During the community attachment process, the students were introduced to the community leaders and other relevant structures upon entry into the community. In their daily community activities, students worked hand in hand with the communities in identifying, prioritizing and addressing community challenges. Students reported that community involvement was critical in solving their health challenges and brought about their participation and ownership which is important for sustainability.

“*We realized that in working with communities it is very crucial to involve the community, you look at what the community considers as a problem so you begin developing an idea to intervene where the community recognizes that it is really a problem affecting them. So, you discuss with them the potential interventions, they tell you their suggestions and you improve their suggestions into something very important, otherwise the intervention will die out; we really appreciated that*.” (FGD Science Students)

c) *Innovative use of available resources.*

In their attachment, students did not have funds for the interventions to address identified challenges, but were expected to use the available local resources in the community. Students reported that they were able to design innovative and sustainable solutions using the available community resources.

“*We went with nothing, with only our heads, and with prior information that OHCEA does not fund anything, I mean whatever intervention that we were going to do, you must use available local resources. At the beginning it was really hard to come up with any intervention without fund. Traditionally when you think of any intervention you first put funds on table, but we were able to put interventions that were really fundamental to the communities without any funds. By doing this, we used what the community would sustain, even without our presence”* (FGD Students Science)

Come up with some useful innovations.

“…*like one time there was a classroom they visited and when they reached that classroom it was full of bats….. They told us the bats do carry germs that cause diseases like marburg. The students using local herbs, came up with a concoction that was able to repel bats when it was smeared in the ceiling of the houses rather than using chemicals (which are eco-unfriendly) to kill them*.” (IDI DVO Kasese)

### Students’ contributions to communities

In all the community interviews, students were appreciated for being very instrumental in addressing community challenges. Community members’ views on the students’ contributions have been categorized into; a) participation in health promotion activities such as health education, sanitation and hygiene, b) enhanced interaction between the communities and their leaders, c) stimulated One Health practice in the communities, d) Committed, caring and compassion.

- *Value in student participation in health promotion activities.*

The students were widely recognized and appreciated by the communities for their engagement in health promotion activities in homes, schools, markets and other communal places, creating awareness among the communities on how to prevent various diseases. They conducted health education sessions which covered various topics including the interconnectedness of animal, human and environmental health.

*“We benefited. We came to learn that there are diseases that can affect both animals and the human beings. These diseases can kill both the animals and human beings.”* (FGD Hima Slaughter Slab operators)

In addition, students were reported to implement sanitation interventions in schools and markets including hand washing facilities, clearing bushes, burning rubbishes, improving drainage systems, cleaning markets and abattoirs, thus improving sanitation standards.

*“The students helped us a lot, they health educated us on health matters, they also participate in cleaning the area, there is a pit at the abattoir they found when it had blocked and they worked with us to dig another pit and filled it with stones, we are generally clean when the students are here. They also gave us advice with regards to good hygiene of the place”* (IDI Tender owner Hima Slaughter Slab*)*

*“They dug two pits down there [at the slaughter slab], they slashed here around the slab and around the toilet, and also cleaned the market. They went to Kisongola primary school and made a water tippy-tap for them,… you know the cows were drinking from* the tap but they fenced the tap.*”* (FGD Slaughter Slab operators)

Also students participated in improving waste management in the markets, by teaching the vendors on how to recycle the waste into briquette’s. Recycling waste into briquettes offered the communities with a number of benefits; improving environmental sanitation, environmental protection and creating an income generating activity for the vendors.

*“… We made briquettes by recycling the waste. At first when we developed the intervention, we mainly concentrated on the waste but when we continued writing the concept, other things came; the economical part, the environmental bit, because the process could also reduce on the deforestation because it reduces on the quantity of charcoal. Also, on the economical bit of it, most women who were working in the market …would sell the briquettes and get some money to cater for their household expenses which they appreciated.”* (FGD Arts Students)

Through their work, the communities reported that students challenged communities and caused visible change and impact in the community for instance in promoting positive attitude towards sanitation. For instance, while a cholera outbreak affected one Kasese district, respondents reported that students work was believed to have contributed in controlling outbreaks in one of the town councils where they were attached.

“*Generally, there is a bit of change of attitude by the community members in as far as sanitation is concerned, because where they found no toilets, they encouraged those people to construct to ensure that they have latrines and so at least sanitation has improved and this has led to reduced episodes infections like cholera and others. For example, when the district was hit by cholera recently, Hima town council was safe. We didn’t have any occurrence of cholera infection.”* (IDI Town Clerk Hima Town Council)

b) *Enhanced interaction between the communities and their leaders.*

Students’ presence in the community created a platform for community members to engage with each other and their leaders. This was appreciated by the community members as it was seen as an opportunity for holding their leaders accountable. Students were also reported to be an effective linkage between the district officials and community members through which their health challenges were reported to the district administration.

“*The students had an opportunity* to *present their work they had done in the community to an audience consisting of the district officials, town clerk, staff and members of councils. They presented what they found in the community, what was done and what is expected of the town council/district, so that the community/town council/district owns it*.” ( IDI Animal Husbandry Hima-Field Mentor)

Students also created awareness regarding potential stakeholders and corporate social responsibility. Students were reported to consult and involve organizations and companies, and reminded them of their role in the community.

“…. *if we have Hima Cement factory in Hima town council, how do they help the community? Do they put there anything, do they give them water, do they open up some roads, how do they contribute to the people, how could people gain from them*?” (IDI Animal Husbandary Hima-Field Mentor)

c) *Stimulated appreciation of One Health practice in the community.*

The students’ way of work challenged community members including the district officials by demonstrating potential efficiency and effectiveness in collaborative efforts. Communities appreciated the One Health approach based on what the students’ way of work, where they exhibited collaboration, team work and respect for each discipline, unlike where they work in siloes. They said that lessons from experiences with students in the communities guided a multi-sectoral response against a cholera outbreak in the district.

“*When we had the recent cholera outbreak, here we had to awaken people, basing on the concept we had from OHCEA, and the fight of cholera it was everyone on board, the LCs were on board, the business community we called them that the strategic market is going to be affected and our business is going to be affected, an outbreak is going to affect the economic activity , so we had to sit with them and agreed on how they were going to improve and we saw things working out, unlike the previous days when it was for the health workers alone*.” (IDI District Health Officer-Field Mentor)

Communities also realized that there are things they should do for themselves, than relying on government.

“*One of the things was that they realized that certain things can be addressed using the available resources other than relying on the government, thinking that it is the government which is going to do everything, they realized that we can address certain things using the resources that are available within us*.” IDI Laboratory-Field Mentor)

d) *Students were committed, caring and compassionate.*

Their work in the communities was greatly valued. With a great desire to impact on communities through changing their attitudes, students were reported to be a committed team that loved what they were doing. They were reported to be responsible and did not shun any kind of work. One KII said that students loved their work and were willing to demonstrate to communities the desired practices.

*“... I witnessed that they were doing very good work. They talked about protection of rivers or water bodies and you could see that they are educating people with a lot of care and love and showing them that it is all about them (community), it is for their benefit that they must do this*.” IDI Town Clerk Hima Town council.

### Challenges faced by students

One of the major challenges reported was related to inadequate funding. While students were encouraged to be innovative and use available resources, they noted that lack of funding limited the extent of the appropriate and sustainable interventions they could implement in the communities. The students felt that if some funds were available, they would have come up with strong and more meaningful solutions than using inferior materials.

*“…We are looking at sustainability and we are just digging up something, no concrete, I think if we were provided with some little cement and you construct a very nice pit, permanent because it will rain and the whole thing is filled with sand, so what next? But if it’s well constructed with concrete, it can create an impact on the whole community and they even make an effort to keep it clean****”*** (IDI Science Student)

Lack of community ownership of interventions was also a concern. It was reported that some community members refused to participate because they wanted to be paid. Such lack of community participation indicates less or no ownership of interventions. The need for payment by some community members before they could to engage in their own community solutions threatens sustainability mechanisms of any interventions the students implemented. Relatedly, community respondents were concerned about the sustainability of the interventions given the short period of attachment because students would leave the communities immediately after implementing the interventions. This raised concerns regarding limited lessons and follow up with some interventions which communities wouldn’t maintain on their own in the long run.

“…*, like issue of sustainability, something has been done, how is the follow up going to be done to ensure sustainability , to show that this is what we are doing and there after what happens? But we need an intervention to be done and people learn from that intervention and people take on that intervention continuity to see that at least there is a change in life, or in the lives of the community, but now the challenge is, after making all this how do we ensure continuity of these services when you leave*” (IDI District Veterinary Officer/Field Mentor)

There was also a challenge of language barrier, with some students and community members failing to communicate which compromised the community engagement process to some extent.

The issue of culture was also raised to affect uptake of what the students sensitized the communities about in terms of preventing diseases. Some cultural practices promote transmission of diseases. For example, it was reported that the *Balalo* (cattle keepers) love their animals so much that they don’t believe they can be of risk to their health. They find it normal to share a house with their animals or drink uncooked milk from them.

*“Ok for the Balalo they got annoyed because for them you cannot separate them with their animals, they wouldn’t mind seeing an animal sharing with them a saucepan. Some of them were not happy with the changes as a result of the interventions of these students”* (FGD Hima Town Council)

## Discussion

This study explored the students’ gains, experiences and their contribution to solving communities’ challenges during a community based interdisciplinary training. The students’ appreciated the training citing skills gained in communication, team work and collaboration. They appreciated that each discipline contributes uniquely and meaningfully towards achieving health in the community. Students also reported a feeling of gratitude and accomplishment. They felt that they had made a positive impact to the community by coming up with interventions to some of the challenges that the communities were facing. The communities learnt and appreciated the concept of One Health from the students. They appreciated the students’ contribution such as improving sanitation and hygiene in schools, slaughter areas and markets. In addition, students raised awareness about general disease prevention and health promotion. They were also reported to exhibit a spirit of team work and collaboration in addition to showing love and commitment to their work in the communities. Students also enhanced interaction between communities and their leaders. However, students encountered some challenges such as language barrier, less community involvement and ownership of the interventions which affects sustainability.

Students embraced the spirit of team work, collaboration and appreciated the relevancy of each discipline’s contribution towards achieving health in the community. This is in line with Frenk’s [1] and Stroud’s [17] recommendation of multidisciplinary and professional interdependence training for the future workforce to be able to form effective teams across disciplines/sectors and cause a transformative change [1]. Interdependence is a pre-requisite for One Health, an approach that recognizes multi-sectoral collaboration locally, regionally and internationally to address health challenges. This multidisciplinary training in the OHI has proved that these One Health competencies can be fostered among a future workforce. Indeed, in addition to their good technical discipline or sector-specific skills, workers also need to be prepared with a collaborative mindset that allows them to organize and maximize their technical skills with other professionals [23]. Thus, a One Health workforce is the sum of its parts, and that collaborative work requires each individual worker to have [1] technical skills and competencies to work well within their own discipline and sector, [2] cross-sectoral skills and competencies to work collaboratively across sectors, and [3] a supportive institution to enable their collaborative work [23]. Bringing together these multidisciplinary student teams to learn, plan and innovate together, fosters the cross-sectoral skills and competencies of collaboration across disciplines/sectors in addition to technical skills and competencies in their own discipline. This provides an environment for partnership which allows multi-sectoral/multidisciplinary collaboration i.e. One Health approach with ability to mobilize and respond to public health challenges [23].

In the same way, the multi-disciplinary community based attachment provided an opportunity to students to innovate. For example a group came up with a bat repellent using the concoctions from the local herbs-which is more environmental friendly than fumigating with chemicals. Exposing students to the realities of health challenges that communities face, makes them more likely to become advocates for change [26, 27] and thus acts as a stimulus for innovations. Thus, the multi-disciplinary training exposure has great potential to expand scientific knowledge and innovation to address public health challenges and thus improve the longevity and quality of life for millions of people. This is because of marked synergy between the different disciplines in achieving human, animal and environment health [28]. As observed in OHI, the foundational technical skills allow students/workers to contribute to niche aspects of the challenges [23]. For example, multi-disciplinary/scientific collaboration between the veterinary and the medical community resulted into the development of the First Balloon Expandable Coronary Stent [28].

The students were taught One Health theoretical courses, which include leadership and management, and equipped with communication, teamwork and collaboration skills. These courses are in line with what Frankson and inter-professional expert panel referred to as the One Health core competencies [29, 30] which are critical needs for successful collaborative efforts. These competencies suggest similar needs for collaborative efforts. By applying One Health core competencies during classroom and field-based training, as was done in the OHI, we can change the way graduates/future workforce see the world, allowing veterinarians, medical professionals, nurses, pharmacists and environmental health practitioners to see their part in the greater system of disease prevention [23].

Community based training is one of the identified institutional capacities important in One Health training [16, 18]. It was a major component in the OHI because it provides an opportunity for experiential learning by integrating education and practice in the learning process [19] fostering competency acquisition. In addition to benefiting students, community based education is meant to benefit communities in terms of providing a service to underserved communities and, hopefully, to affect student career choices [31–33]. In the OHI field attachment, communities appreciated students’ contribution ranging from interventions such as improving sanitation and hygiene in schools, abattoirs and markets as well as creating awareness about diseases prevention and health promotion. In addition, students were also reported to pass on cross-sectoral skills to the community members, having a multiplier effect on student training in a community based approach. The benefits of the OHI student attachment to the community makes community based education a ‘win win’ program for both students and communities [25].

However, there were challenges that students faced such as lack of community engagement linked to high community expectations that were not fulfilled which can threaten community ownership and sustainability of the interventions. This calls for what Williams and colleagues suggested [34]- the need for a more balanced partnership where the community is consulted in planning a health programme that is relevant to that community’s particular needs. The need to work in a partnership while planning for a community based training is very critical so that the roles and expectations of each party are clarified. As was raised by Mbalinda and colleagues [35], the long-term success or failure of a community based training programme rests on the nature of the agreement between the stakeholders. Indeed, from the interviews, students reported that community involvement was critical in solving their health challenges and attracted their participation and ownership which is important for sustainability. For this community involvement to be successful, benefiting both parties and fostering ownership and sustainability, it should start with the planning process of the activities and a dialogic relationship between the two partners [31, 36]. Overall, community based education has been found to increase service delivery to remote and rural areas through improved retention of workers [19, 33], by improving awareness of community values as well as increases trainees’ interest in uptake of careers in rural practice [24, 37].

Globally, there are similar initiatives like OHI. In North America the Duke University is home to the Duke One Health Training Program and Duke One Health Research Team. The program involves 3.5 weeks of intense graduate training in One Health (four courses) and often engages international professional and graduate students from 10 or more countries [38]. This a certificate course. In addition, there are many more similar training programs as reported in the paper by Stroud and colleagues [17]. Further, the Students for One Health (SOH) groups have formed in universities all over the world to further One Health education- implementing local discussions on One Health topics as well as engaging communities by holding One Health ‘clinics’ [17]. Elsewhere similar initiatives are being planned. A recent conference of the European Organisation representing medical doctors, dentists, pharmacists and veterinarians and their students’ on “Implementation of One Health in Undergraduate Education”, the importance of developing a One Health culture in universities to enhance One Health collaboration in practice was highlighted. An update of the curricula to encompass and promote interdisciplinary education, train educators to encourage more interaction among students of different backgrounds and launch and support joint initiatives to further implement the One Health approach was agreed upon [39].

Despite these efforts, One Health initiatives are faced with challenges. Firstly, there is no guidance on a common understanding and implementation of One Health in practice, as well as promoting interdisciplinary collaboration. Secondly, educators need training and encouragement to consider a different model of teaching that facilitates interdisciplinary interaction and education [39]. There is lack of One Health practitioners trained to facilitate and coordinate collaborations within One Health projects and few and inefficient One Health training programs due to lack of academic and institutional support for One Health [40]. Further, a number of academic, government and private partners are involved in One Health training and research opportunities. But it is not clear if the One Health ‘movement’ will evolve into a mainstream ‘discipline’ or remain an ‘approach’ to global public and planetary health as some One Health leaders advocate [17]. There are arguments against the approach because they are concerned that the quality of training that specialties provide [17]. Some solutions have be suggested to foster the start of One Health such as; improving political support and access to funds, and enhancing the educational and training opportunities for One Health practitioners [40]. Therefore, initiatives like the one in OHI (students in different disciplines learning together and the community based approach) are steps in the right direction in fostering OH implementation in academia and overall implementation.

### Strengths and limitations of the study

The OHI uses a unique approach to field-based training, in that it is multidisciplinary in nature, while most community based education programs have been conducted mainly by health science students or one discipline [31–33]. In addition, this study captured both the students and communities’ views and gains from the OHI attachments. This is in line with the ideal community-based training program being a ‘win-win’ programme as it provides both the training institution and the service site with additional resources [25]. However, the study had limitations; being qualitative in nature, the findings cannot be generalizable. In addition, the lack of pre-experience interview to draw a contrast with the cited post-experience interviews.

## Conclusion

The OHI was an attempt to put in practice the recommendations of global movements and the Commission on the Education for health professionals for the twenty-first century, by redesigning teaching for the future workforce from silo based to multidisciplinary and professional interdependence manner. The experiences of students and communities indicate that the OHI training caused attitudinal change among students to view the need for professional collaborations as requirement for effective solutions of current and future health challenges. Such training should be promoted in similar settings like Uganda. One question is what does it take to institutionalize and scale up this kind of training which should be explored in future? We also recommend further studies including follow-up with the students to ascertain what this experience has meant to them in their professional development.

## Data Availability

The data that support the findings of this study are available from the main author, however restrictions apply to the availability of these data, given that study only utilized qualitative data. ln general, it is difficult to fully de-identify qualitative data and difficult to interpret without an understanding of the context. In addition, there were local terms (commonly spoken local language) in the text. As a result, this makes it hard to share the source data. Data are however available from the authors upon reasonable request and with permission of OHCEA Project.

## Abbreviations

IDIs: In-depth interviews
FGDs: Focus group discussions
OHCEA: One Health Central and Eastern Africa
OHI: One Health Institute
OHWF: One Health Workforce
